# Inhibition of Pre-mRNA Splicing by a Synthetic Blom7α-Interacting Small RNA

**DOI:** 10.1371/journal.pone.0047497

**Published:** 2012-10-29

**Authors:** Marlies Löscher, Markus Schosserer, Eric Dausse, Kiseok Lee, Paul Ajuh, Regina Grillari-Voglauer, Angus I. Lamond, Jean-Jacques Toulmé, Johannes Grillari

**Affiliations:** 1 Department of Biotechnology, BOKU - University of Natural Resources and Life Sciences, Vienna, Austria; 2 INSERM U869, European Institute of Chemistry and Biology, Pessac, France; 3 University of Bordeaux, Bordeaux, France; 4 School of Life Sciences, Welcome Trust Biocentre, University of Dundee, Dundee, United Kingdom; 5 ACIB, Vienna, Austria; 6 Evercyte GmbH, Vienna, Austria; International Centre for Genetic Engineering and Biotechnology, Italy

## Abstract

Originally the novel protein Blom7α was identified as novel pre-mRNA splicing factor that interacts with SNEV^Prp19/Pso4^, an essential protein involved in extension of human endothelial cell life span, DNA damage repair, the ubiquitin-proteasome system, and pre-mRNA splicing. Blom7α belongs to the heteronuclear ribonucleoprotein K homology (KH) protein family, displaying 2 KH domains, a well conserved and widespread RNA-binding motif. In order to identify specific sequence binding motifs, we here used Systematic Evolution of Ligands by Exponential Enrichment (SELEX) with a synthetic RNA library. Besides sequence motifs like (U/A)_1–4_ C_2–6_ (U/A)_1–5_, we identified an AC-rich RNA-aptamer that we termed AK48 (Aptamer KH-binding 48), binding to Blom7α with high affinity. Addition of AK48 to pre-mRNA splicing reactions in vitro inhibited the formation of mature spliced mRNA and led to a slight accumulation of the H complex of the spliceosome. These results suggest that the RNA binding activity of Blom7α might be required for pre-mRNA splicing catalysis. The inhibition of in-vitro splicing by the small RNA AK48 indicates the potential use of small RNA molecules in targeting the spliceosome complex as a novel target for drug development.

## Introduction

The protein coding information in eukaryotic organisms is split by intronic sequences containing regulatory elements and microRNAs. However, these introns have to be removed during synthesis of mRNAs by pre-mRNA splicing. This process is performed by the spliceosome, a large multi-protein machinery consisting of four small nuclear ribonucleoprotein particles and more than 100 different proteins that assemble dynamically in a step-wise manner on the pre-mRNA [1]. One distinct sub-complex associated with the spliceosome is the CDC5L/SNEV^Prp19/Pso4^ complex, which consists of SNEV^Prp19/Pso4^, CDC5L, PLRG1, SPF27 (BCAS2) and Hsp73 forming the core complex, while also additional proteins are associated [2], [3]. This complex is necessary for the catalytic steps of pre-mRNA splicing since its immunodepletion results in blocking of pre-mRNA splicing in vitro [4], [5]. Similarly, inhibition of the interaction between different subunit members like CDC5L and PLRG1 [4] block splicing, whereby disruption of the multimerisation of SNEV^Prp19/Pso4^ even blocks spliceosome assembly [5].

Recently we identified Blom7α as another protein, which is associated with this complex by direct interaction with SNEV^Prp19/Pso4^ [6]. Besides its role as essential mRNA splicing factor [5], SNEV^Prp19/Pso4^ is differentially regulated in replicative senescence of human endothelial cells [7] and extends their replicative life span when overexpressed [8]. It also plays a role in DNA damage repair [9], [10] and interacts with the proteasome [11]. Furthermore, SNEV^Prp19/Pso4^ is an essential protein in early mouse development [12], presumably due to its role as essential pre-mRNA splicing factor [5].

Previously, we reported that Blom7α also is involved in pre-mRNA splicing, since besides its co-localization and co-precipitation with other known splicing factors, its addition to nuclear extracts increases the splicing activity in vitro, and co-transfection with splicing reporter minigenes alters the pattern of alternatively spliced variants [6]. Sequence analysis identified two heteronuclear ribonucleoprotein K (KH) domains in the N-terminal half of Blom7α. Interestingly, at least two further splice isoforms of Blom7α [GenBank ID: AAM51855.1], termed Blom7β [GenBank ID: AAM51856.1] and Blom7γ [GenBank ID: AAM51857.1], of yet unknown function exist, which share the N-terminal KH domain [6].

Here we report the characterization of the RNA binding activity of Blom7α and show that Blom7α co-localizes with RNA in cells. Furthermore, we describe the identification of a splicing inhibitory RNA aptamer, termed AK48 (Aptamer KH-binding 48), which was selected against the KH domains of Blom7α by Systematic Evolution of Ligands by Exponential Enrichment (SELEX).

Aptamers have been developed in the early 90's [13]–[15]. These structured DNA, RNA or modified oligonucleotides are identified after iterative cycles of selection/amplification through SELEX from a random oligonucleotide library. Aptamers were successfully selected for a wide range of targets (proteins, nucleic acids, peptides, small molecules, cells,…) and were shown to display both high affinity and specificity [16], [17]. Aptamer-based tools are a promising alternative to monoclonal antibodies in many applications [18], [19] including molecular imaging [20].

## Results

### Blom7α consists of two KH domains which co-localize with RNA

**Figure 1:**
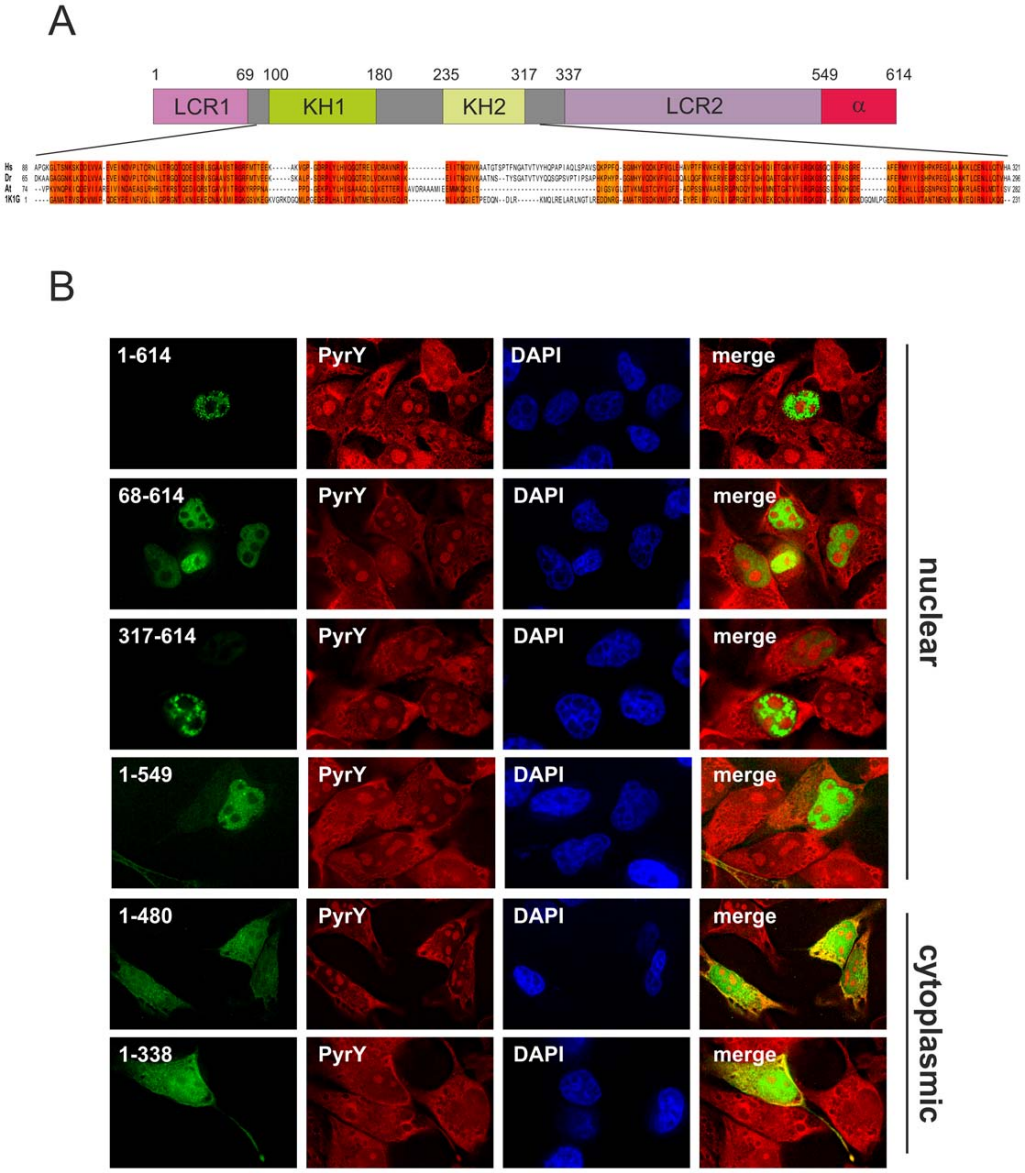
Nuclear retardation of Blom7α is driven by amino acids 480–549. (**A**) Protein domain architecture of Blom7 isoforms. LCR1: low complexity region APG-rich; KH1: KH domain (RNA binding domain) 1; KH2: KH domain (RNA binding domain) 2; LCR2: low complexity region PS-rich; α: α-specific C-terminus. Below a multiple sequence alignment of the KH domains of Blom7α with its *D. rerio* and *A. thaliana* orthologues, as well as a known KH domain containing protein is shown. The positional conservation is indicated in colors. (**B**) Localization of GFP-Blom7α truncation mutants in Hela cells.

We recently identified Blom7α as novel alternative splicing factor [6]. By performing a PSI-Blast search and a multiple sequence alignment of human Blom7α with its *Danio rerio* and *Arabidopsis thaliana* orthologues, as well as with a known KH domain containing protein [PDB ID: 1K1G], we identified two highly conserved KH domains within Blom7α (Fig. 1A).

In the literature, KH domains are described as being important for the interaction of proteins with RNA or DNA [21]–[23]. We therefore wanted to elucidate the cellular localization of diverse GFP-Blom7α truncation mutants, which were either completely lacking the region containing one or both KH motifs or consisting of these domains alone.

As expected, full length Blom7α mainly localized to the nucleus of Hela cells, as we already described for the endogenous protein [6]. This was also true for all mutants containing the region between amino acids 480 and 549. However, mutants lacking this part and containing the KH motifs localized to nucleus, as well as cytoplasm, as was already described for Blom7γ, which is composed of the KH domains and the γ-specific C-terminus alone [6]. The cytoplasmic staining fully co-localized with RNA counterstained by Pyronin Y (Fig. 1B). From this we conclude that the region between amino acids 480 and 549 is necessary for full retention of Blom7α in the nucleus. However, if this part is missing, Blom7α is additionally present in the cytoplasm, suggesting a co-localization with RNA, possibly due to a direct interaction of the KH domains with RNA.

### In vitro selection of aptamers binding to Blom7α and its KH domains by SELEX

**Figure 2:**
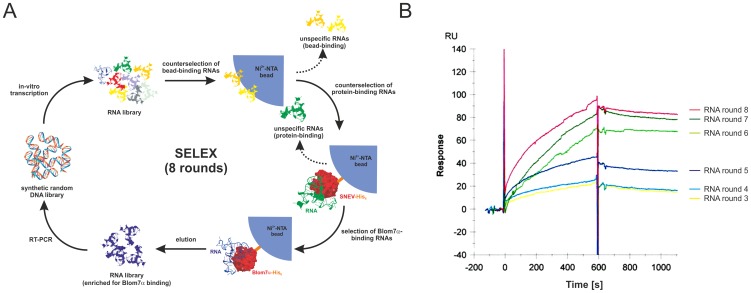
Enrichment for Blom7 binding RNA. (**A**) Outline of the SELEX procedure. (**B**) Result of SPR analysis using enriched RNA (2 µM) specific to Blom7-KH on immobilized Blom7α wt.

In order to further characterize Blom7α, we decided to identify a general Blom7α RNA binding sequence, especially for the highly conserved KH domains. Therefore, we performed SELEX of full-length Blom7α (Blom7α wt), or its N-terminal half consisting of the KH domains alone (Blom7-KH) using a synthetic RNA library of 30 random nucleotides with 8 rounds of selection including counter selection steps for increased affinity as outlined in Fig. 2A. We decided to use Blom7-KH instead of the two KH domains separately, because these domains are described to act often in concert to mediate specific RNA-protein interactions [23]. In order to monitor the increase of the binding affinity of selected RNA pools during the progress of SELEX and to confirm the interaction between the selected RNAs and proteins, Surface Plasmon Resonance (SPR) analyses were performed after each round of selection.

Then, RNA was injected over the functionalized surface and resulted in binding as shown by increasing response units (RU) values (Fig. 2B). Non-specific binding was subtracted using blank runs on a surface not loaded with protein and on a surface loaded with streptavidin as non RNA-binding control.

Surprisingly, no dissociation of the RNA-protein interaction was detected indicating a long-lived complex and consequently a high equilibrium binding constant. Dissociation of RNA was achieved only by applying several pulses of 10 mM NaOH, while all other regeneration agents tested failed to disrupt RNA-protein interactions.

As could be concluded from the sensorgram, the k_on_ (association) of the selected RNAs assembling on the proteins is very low, and in contrast the k_off_ (dissociation) is very high. This leads to the hypothesis that there is a slow RNA-protein-complex formation, but once the complex is assembled and in the correct conformation, it is very stable.

### Identification of a common binding motif and specifically binding aptamers

As SPR analysis confirmed the interaction of selected RNAs with Blom7α, RNA libraries from the last round of selection were reverse-transcribed and TOPO-cloned. The screening of positive colonies resulted in 59 Blom7α wt specific clones, and in 69 positive Blom7-KH specific clones, which were subjected to sequencing.

**Figure 3:**
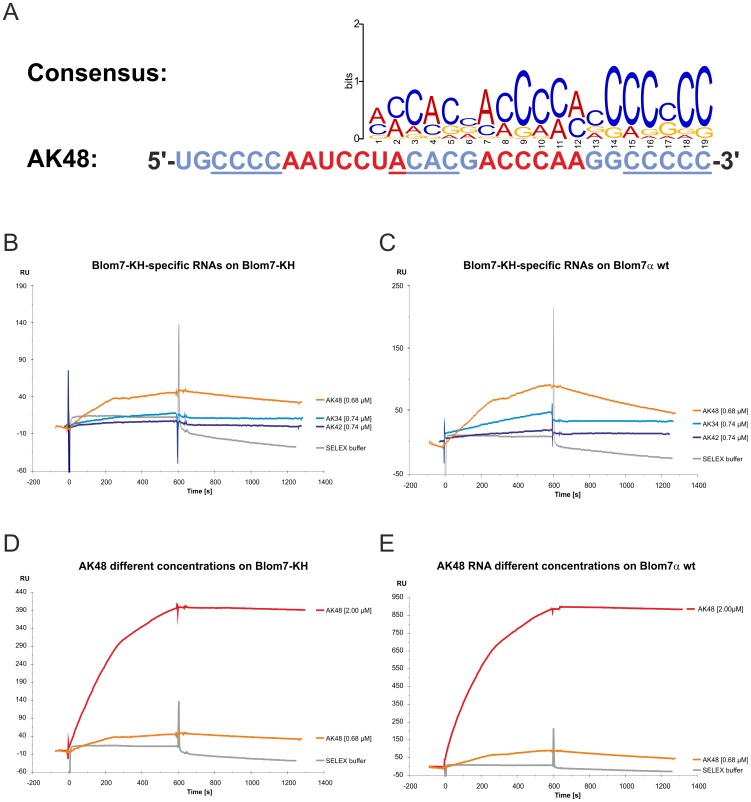
Selected RNA-aptamer AK48 binds to Blom7α . (**A**) Consensus sequence of putative binding motif of all selected sequences (Blom7α wt and Blom7-KH) in alignment with AK48 (red: putative binding motif, underscore: AC-rich part and poly-C stretch. (**B, C**) Result of SPR analysis using AK48 (0.68 µM) and non/weak binding KH-specific RNAs (0.74 µM) on immobilized Blom7α wt (**B**) or Blom7-KH (**C**). (**D, E**) Result of SPR analysis using AK48 (0.68 and 2 µM) on immobilized Blom7α wt (**D**) or Blom7-KH (**E**).

The obtained sequences were manually aligned and the following common binding motif was identified: **(U/A)_1–4_ C_2–6_ (U/A)_1–5_**. Furthermore, we computed a consensus sequence, which clearly displays an abundance of A/C rich sequences and poly-C stretches at the 3′-end (Fig. 3A).

8 clones containing characteristic elements of the putative motif were selected for retesting of the isolated sequences. Three of the eight clones showed clear binding in SPR experiments (data not shown). Comparison of two other candidates with AK48, the aptamer with the highest response selected against Blom7-KH, demonstrates clearly the higher binding affinity of AK48. (Fig. 3B, C). Again, very slow association and nearly no dissociation was observed. Interestingly, the binding of the selected KH-specific aptamers resulted in higher RU levels on Blom7α wt than on Blom7-KH (Fig. 3B, C), possibly because full-length Blom7α contains additional domains in its C-terminal region helping to stabilize the interaction with RNA.

AK48 was selected for further analysis and subjected in concentrations of 0.68 and 2 µM to SPR. As expected, with 2 µM a higher response as with 0.68 µM was observed (Fig. 3D, E). However, during the analysis of the binding properties of AK48 against Blom7α wt and Blom7-KH, the sensorgrams could not be properly fitted to a 1∶1 model preventing the determination of the rate constants k_on_ and k_off_ (data not shown). Furthermore, the association phase has an unusual shape, which might indicate a more complex binding mechanism (bi-phasic, cooperative or binding of two aptamers) and is possibly caused by the two KH domains within Blom7α.

The sequence of AK48 aligned with the putative motif is shown in Fig. 3A.

### Confirmation of binding of AK48 to Blom7α by EMSA

**Figure 4:**
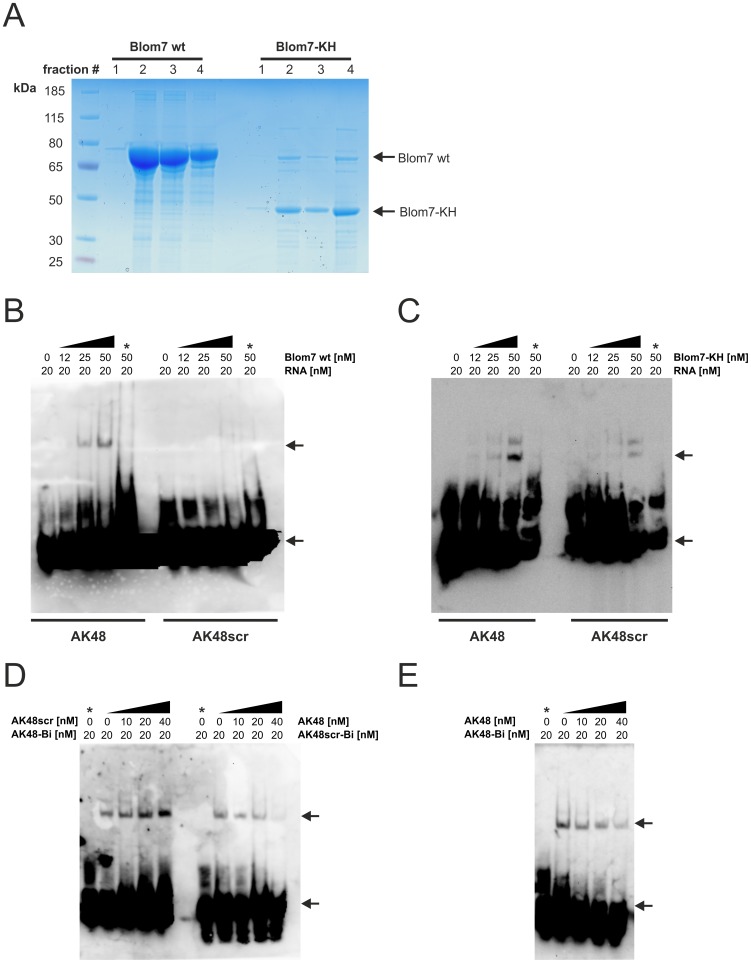
Confirmation of binding of AK48 to Blom7α by EMSA. (**A**) Coomassie-stained PAGE-gel after the purification of recombinant Blom7 wt and Blom7-KH. (**B**) EMSA of increasing concentrations of Blom7 wt (0–50 nM) incubated with constant amounts of biotinylated AK48 or AK48scr (20 nM). The positions of the bands corresponding to free RNA or RNA-protein complexes are indicated by arrows. Lanes containing additionally 50 mM unlabeled AK48 or AK48scr are indicated by an asterisk (*). (**C**) EMSA of increasing concentrations of Blom7-KH (0–50 nM) incubated with constant amounts of biotinylated AK48 or AK48scr (20 nM). The positions of the bands corresponding to free RNA or RNA-protein complexes are indicated by arrows. Lanes containing additionally 50 mM unlabeled AK48 or AK48scr are indicated by an asterisk (*). (**D**) EMSA of 50 nM Blom7 wt incubated with a mixture of 20 nM biotinylated AK48 or AK48scr and increasing amounts of unlabeled RNAs (0–40 nM). The positions of the bands corresponding to free RNA or RNA-protein complexes are indicated by arrows. Lanes containing no protein are indicated by an asterisk (*). (**E**) EMSA of 50 nM Blom7 wt incubated with 20 nM of biotinylated AK48. After 10 min increasing amounts of unlabeled RNA (0–40 nM) were added and incubated for additional 10 min in order to investigate the dissociation rate. The positions of the bands corresponding to free RNA or RNA-protein complexes are indicated by arrows. Lanes containing no protein are indicated by an asterisk (*).

In order to verify the interaction between Blom7α and AK48 with an independent method, we performed electrophoretic mobility shift assays (EMSAs). Therefore, we used recombinant HIS-tagged Blom7α wt and Blom7-KH, expressed in *E. coli*. After the purification, fractions 2–4 showed clear bands at the correct heights with minimal degradation products (Fig. 4A), and were therefore pooled for subsequent experiments.

As expected, in EMSA we detected a band-shift when using 20 nM biotinylated AK48 and increasing amounts of Blom7α wt (Fig. 4B), or as well with Blom7-KH (Fig. 4C). Surprisingly, also AK48scr, RNA with the same base composition and length but scrambled sequence, shows weak interaction with Blom7α wt, as well as with Blom7-KH. However, when the protein was pre-incubated with a high molar excess of unlabeled AK48 or AK48scr, no band shift at the expected height was observed (Fig. 4B and 4C, lanes with asterisk). Still this prompted us to test, if affinity of Blom7 wt for AK48 is higher than for AK48scr by offering either labeled AK48 together with increasing amounts of AK48scr or vice versa. Thereby, we clearly see higher affinity towards AK48 (Fig. 4D).

Finally, since we were not able to remove the RNA from Blom7α or the Blom7-KH domain in the SPR-assays above, we tested if 10 minutes pre-incubation with the biotin-labeled AK48 could be competed out by unlabeled RNA. Indeed, not even a 4-fold molar excess of unlabeled AK48 (lanes 2–5) could completely compete out biotinylated AK48 from binding to Blom7 wt (Fig. 4E). This result is in perfect accordance with the SPR-data, where we also observed extremely low dissociation rates.

### AK48 inhibits pre-mRNA splicing in vitro

**Figure 5:**
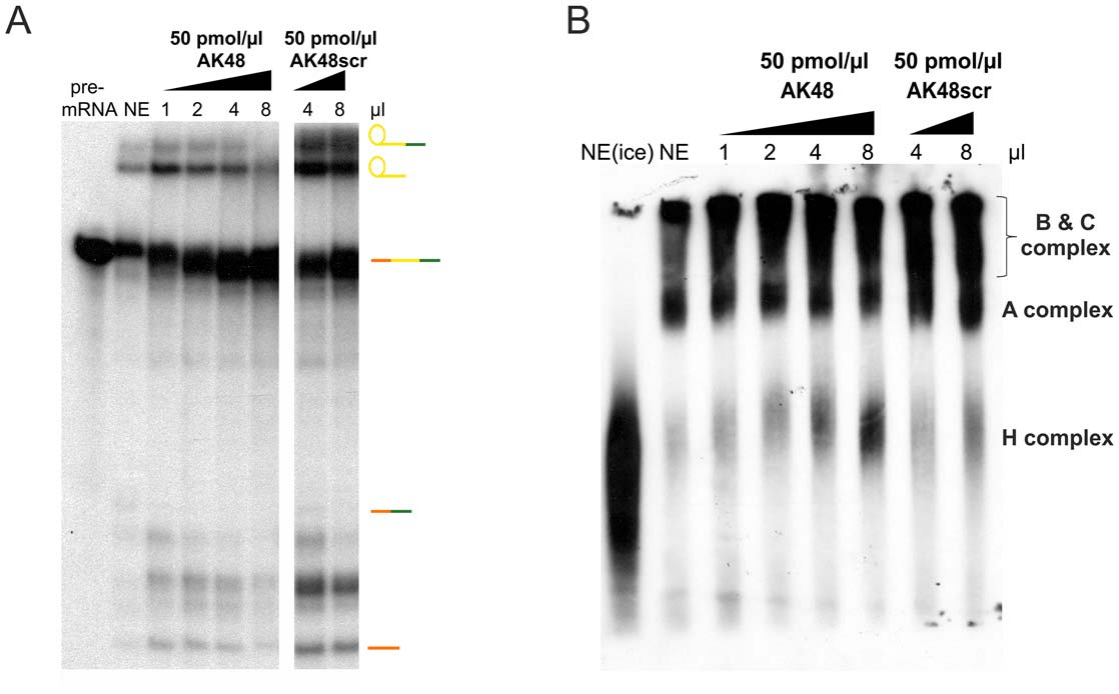
Influence of AK48 RNA on pre-mRNA splicing. (**A**) Inhibition of *in vitro* splicing reaction by AK48. Splicing assay using uniformly labeled capped pre-RNA incubated with nuclear extracts (NE) and increasing amounts of AK48, respectively AK48scr (scrambled). (**B**) Addition of increasing amounts of AK48 leads to an accumulation of the H-complex.

Since we described previously that addition of recombinant Blom7α to Hela nuclear extracts increased splicing activity in a dose-dependent manner [6], we here tested whether AK48 binding to the KH domains of Blom7α might alter splicing activity in vitro. Indeed, we detected a decrease in lariat-formation and an increase in accumulating pre-mRNA with increasing amounts of AK48, but not with a scrambled control (Fig. 5A). Interestingly, addition of AK48 increased formation of the H-complex, suggesting interference with spliceosome assembly (Fig. 5B).

Although these data suggest that the endogenous RNA binding activity of Blom7α might be essential for its function during the splicing reaction, we cannot rule out that AK48 interacts in-vivo preferably with another unknown protein involved in pre-mRNA splicing (possibly also containing KH domains), which causes the inhibition of pre-mRNA splicing independent of Blom7α.

## Discussion

In order to further characterize the novel pre-mRNA splicing factor Blom7α, we here investigated on the ability of Blom7α to bind RNA. Therefore, we performed co-localization studies in Hela cells and found the KH-domain-containing part of Blom7α to be present in the cytoplasm, possibly due to binding of the KH-domains to RNA. Furthermore, we mapped the domain necessary for nuclear retention to a region between amino acids 480 and 549. Since no canonical nuclear localization signal (NLS) is present within Blom7α, the nuclear localization is likely to be driven by interaction with other factors. For instance SNEV^Prp19/Pso4^, a protein predominantly present in the nucleus, was previously shown to physically interact with the C-terminal domain of Blom7α [6].

As a next step, we performed SELEX in order to detect putative RNA aptamers binding to Blom7α. Indeed, RNAs binding to full-length Blom7α, as well as to the KH-domain containing part, were enriched during the progress of SELEX. Thus, our hypothesis that the KH domains of Blom7α are responsible for RNA-binding is strengthened.

KH domains are besides the RNA recognition motif (RRM) the most abundant nucleic acid-binding domains. Their widespread presence in eubacteria and eukaryotes suggests them to be of ancient evolutionary origin. Proteins containing KH domains are involved in the regulation of gene expression at several levels, such as in transcriptional or translational regulation, splicing and mRNA transport, stability, and localization [24], [25]. It was shown that the three KH domains of hnRNP K bind in a cooperative manner to mRNA targets [23], making it plausible, that multiple KH domains like in Blom7α not only increase the binding affinity, but also select for longer nucleotide stretches. Thus, they might be key determinants of selectivity of RNA-protein binding. Different KH domain proteins may achieve affinity and specificity for target RNAs by contacting additional sequences surrounding short pyrimidine-rich core motifs, thus the context surrounding these motifs may play a major role in generating high affinity binding sites.

We observed extremely low dissociation rates of different RNAs in SPR as well as in EMSA, after having bound to Blom7. It might therefore be possible, that Blom7α undergoes conformational changes upon binding to RNA, as was already reported for various other factors interacting with RNA [26]. This conformational change possibly locks the RNA within the binding site of Blom7α, as is indicated by the extremely low dissociation rates, and can only be released upon further changes in protein structure. Such a release might be mediated by protein-protein interactions or post-translational modifications that are still unknown in the case of Blom7α.

As we found Blom7α to be involved in the splicing process, an additional hint for the importance of the found A/C rich motif as common regulatory element is the fact that another nuclear protein (YB-1) binds to an A/C-rich exon enhancer element in CD44 exon v4, which is required for maximal *in vivo* splicing and *in vitro* spliceosome assembly [27]. Astoundingly our identified Blom7α binding-motif is highly similar to this exon enhancer element (CAACCACA) strongly supporting the hypothesis that binding of Blom7α to this or a similar motif is necessary for efficient splicing and spliceosome assembly. However, we cannot rule out yet, that Blom7α is binding RNA as a co-factor forming an RNP and that therefore an endogenous RNA essential for splicing catalysis has yet to be identified. Furthermore, our experiments cannot exclude the possibility that the effect of AK48 on pre-mRNA splicing is solely due to its interaction with Blom7α, but might also come from binding to other proteins involved in that process, possibly containing KH-domains likewise. Finally, although this possibility seems very unlikely, we cannot completely rule out that AK48 interacts with some co-purified factors present in the extract instead of Blom7, as we lack a suitable antibody for supershift analysis.

For RRM (RNA recognition motif), the most abundant and best characterized RNA binding module, the beta-sheet is the primary surface for RNA recognition, while additional contacts mediated by N- and C-terminal residues or loops are important in determining substrate specificity [28], [29]. Interestingly, the helical surface of the RRM fold is often involved in protein-protein interactions, while the beta-sheet platform on the opposite side mediates RNA binding. Blom7α might show a similar mechanism: N-terminal KH domains mediate protein-RNA interaction via beta-sheet surfaces, while the C-terminal α-specific tail triggers binding of regulatory proteins, as was already confirmed for SNEV^Prp19/Pso4^ [6].

In conclusion, small synthetic RNAs as general inhibitors of splicing catalysis might represent the starting point to develop novel therapeutics targeting the process of pre-mRNA splicing.

## Materials and Methods

### Alignment of Blom7 sequences in the region of KH domain matches

In order to identify conserved protein domains within Blom7α, PSI-BLAST [30] searches for significantly similar sequence segments in the non-redundant database (inclusion E-value 0.001) were performed. The similarity searches were started separately with the polypeptide segments 1–230 and 200–614 of human Blom7α protein [GenBank ID: AAM51855.1]. Afterwards, Blom7α from *Homo sapiens* (Hs), as well as highly similar sequences from *Danio rerio* [GenBank ID: NP_997758.1] and *Arabidopsis thaliana* [GenBank ID: NP_566850.3] were aligned with two copies of a part of an already structurally characterized human KH domain protein [PDB ID: 1K1G] using T-Coffee [31]. The alignment was drawn with the help of Jalview [32] (version 2.7).

### Cell culture, transfection, and fluorescence microscopy

HeLa cells were grown in DMEM (Dulbecco's modified Eagle's medium) supplemented with 4 mM L-glutamine and 10% (v/v) fetal calf serum.

Hela were transfected with Blom7 truncation mutants in pEGFP-C1 using Lipofectamine 2000 (Life Technologies). 48 hours after transfection the cells were washed with PBS and fixed for 5 min in 3.7% (w/v) paraformaldehyde in CSK buffer (10 mM Pipes, pH 6.8, 10 mM NaCl, 300 mM sucrose, 3 mM MgCl_2_ and 2 mM EDTA) at room temperature, followed by counterstaining for DNA with DAPI and RNA with Pyronin Y. Microscopy and image analysis were performed using a Zeiss DeltaVision Restoration microscope as described [5], [6], [33].

### SELEX

RNA SELEX was performed as previously described [34]. For a detailed protocol please refer to the Supplementary material available online.

In brief, a synthetic DNA random library (Proligo) of 30 random nucleotides (4^30^_UACG_ = theoretically 10^18^ different sequences) flanked by 5′ and 3′ invariant linker sequences (5′-GTGTGACCGACCGTGGTGC-3′ and 5′-GCAGTGAAGGCTGGTAACC-3′) was amplified by PCR with 3′SL downstream primers (5′-TAATACGACTCACTATAGGTTACCAGCCTTCACTGC-3′) containing the T7 transcription promoter (underlined) and P20 upstream primers (5′-GTGTGACCGACCGTGGTGC-3′) and in vitro transcribed. This RNA pool was used for the first round of selection. All steps of in vitro selection were performed in 100 µl SELEX binding buffer (1× PBS, 1 mM magnesium acetate, pH 7.0) on ice.

10 pmol Blom7 wt-His_6_ and Blom7-KH-His_6_ were coupled to Ni^2+^-NTA beads (Promega) for the 1^st^ round and washed several times with SELEX binding buffer. After two rounds of counterselection on uncoated and His_6_- SNEV^Prp19/Pso4^ coated beads in order to eliminate unspecific binders, the RNA pool was added to Blom7 wt or Blom7-KH coupled beads and incubated for 45 min on ice. Beads carrying protein-RNA complexes were washed with SELEX binding buffer several times and bound RNA was eluted by addition of nuclease-free water and heating for 1 min to 75°C.

Afterwards, RNA candidates were reverse-transcribed and amplified by PCR. Following rounds of selection were performed as before.

### Cloning of candidate RNAs

After 8 cycles of selection against Blom7 wt or Blom7-KH, candidates were cloned using the TOPO TA Cloning Kit (Life Technologies) according to the manufactures' instructions. Plasmids of 100 positive clones of each selection were Sanger-sequenced.

### Surface Plasmon Resonance measurements (SPR)

SPR (surface plasmon resonance) experiments were performed using the BIACORE 3000 (GE Healthcare) system and Sensor Chip CM5 (GE Healthcare) at 23°C.

Activation of the chip surface with a 1∶1 mixture of N-hydroxysuccinimide (NHS) (50 mM) and 1-ethyl-3-(3-dimethylpropyl)-carboimide (EDC) (200 mM) and immobilization of proteins (500 nM in 10 mM NaAc pH 5) were performed following the manufacturer's instructions. Afterwards the chip surface was saturated with ethanolamine (1 M, pH 8.5) and the system primed with SELEX binding buffer or HEPES buffer containing 3 mM magnesium acetate. RNAs were refolded by heating (95°C for 1 min), freezing (4°C for 3 min), and keeping at room temperature for 5 min, diluted (0.68, 0.74 or 2 µM) in SELEX binding buffer and injected (flow rate 10 µl/min, injected volume 100 µl). Unspecific binding was considered by subtracting blank runs on a surface not loaded with protein and on a surface loaded with a non RNA-binding control protein. The association and dissociation rates were monitored for 600 s each. Regeneration of the chip surface was performed using pulses of 10 mM NaOH.

### RNA motif prediction

The computation of the consensus sequence of selected RNAs was done with MEME [35].

### EMSA

Biotinylated versions of AK48 (5′-UGCCCCAAUCCUACACGACCCAAGGCCCCC-3′-Bi) and AK48scr (5′-CACGCACUCACCACGCACGCAUCCGCUCAC-3′-Bi) were synthesized and PAGE-purified by Dharmacon.

Blom7 wt-His_6_ and Blom7-KH-His_6_ were PCR-cloned into pET30A (Qiagen) and transformed into BL21. After purification over Ni-NTA-beads (Qiagen) and dialysis against PBS, fractions 1–4 were loaded onto 4–12% PAGE gels (Invitrogen) and stained with PAGE-Blue (Fermentas) according to the manufacturer's instructions. Fractions 2–4 were pooled and used for subsequent experiments. Quantification of proteins was performed by OD280 measurement and calculation of concentrations by using the specific molar extinction coefficients (41870 for Blom7 wt and 13850 for Blom7-KH).

For EMSAs, RNAs were refolded by heating (95°C for 1 min), freezing (4°C for 3 min), and keeping at room temperature for 5 min. 20 µl reactions were set up in PBS (pH 7.5) containing 1 mM MgCl_2_, 1 mM DTT and 5% glycerol, as well as RNAs and proteins as indicated in the figure legends. Biotinylated RNAs were always added last and reactions were incubated for 20 min at room temperature, if not indicated otherwise. Afterwards the samples were carefully mixed with 5 µl 5× REMSA loading dye (Pierce) and loaded on a 6% polyacrylamide DNA Retardation Gel (Invitrogen). Electrophoresis was performed in 0.5× TBE at 100 V for 90 min at 4°C. The RNAs were transferred to positively charged nylon membranes (Roche) by tank blotting in 0.5× TBE at 400 mA for 40 min at 4°C and crosslinked at 120 mJ/cm^2^ at 254 nm. For the detection of RNAs the Chemiluminescent Nucleic Acid Detection Module (Pierce) was used according to the manufacturer's instruction. Bands were visualized on a Lumi-Imager (Roche) in chemiluminescence mode.

### In vitro splicing assay and native gels

Nuclear extracts used in the splicing assays were obtained commercially from Dundee Cell Products Ltd (Dundee, UK). Splicing assays and analysis of spliceosome assembly were done as described previously [36], [37]. Synthetic RNA was added to the splicing reactions in the concentrations indicated in the figure legends.

## Supporting Information

Supporting Methods S1 **Detailed protocols for SELEX and SPR-analysis.**
(DOCX)
