# Supplementary material for: Inhibition of Pre-mRNA Splicing by a Synthetic Blom7α-Interacting Small RNA
Source: PLoS One. 2012 Oct 29;7(10):e47497. doi: 10.1371/journal.pone.0047497 (PMC3483155; doi:10.1371/journal.pone.0047497)
Supplement: Supporting Methods S1 — Detailed protocols for SELEX and SPR-analysis. (DOCX) [file pone.0047497.s001.docx]

**S1 Supplementary Methods:**

SELEX

A synthetic DNA random library (Proligo) of 30 random nucleotides (4^30^_UACG_ = theoretically 10^18^ different sequences) flanked by 5' and 3' invariant linker sequences (5'-GTGTGACCGACCGTGGTGC-3’ and 5'-GCAGTGAAGGCTGGTAACC-3’) was amplified by PCR using AmpliTaq Gold polymerase (Applied Biosystems) and 3´SL downstream primers 5'-TAATACGACTCACTATAGGTTACCAGCCTTCACTGC-3’ (including the T7 polymerase transcription promoter complementary to the 3' end of the DNA library), as well as P20 upstream primers 5'-GTGTGACCGACCGTGGTGC-3’ (for hybridization to the 5' complement of the library and subsequent transcribtion into RNA). After phenol/chloroform extraction and ethanol precipitation of the PCR products, the in vitro transcription reaction was performed for 2 h at 37°C using the Ampliscribe T7 high-yield transcription kit (Epicentre Technologies). In vitro transcribed RNA was quality controlled on denaturing polyacrylamide / TBE gels. Bands were visualized by UV shadowing, cut out, and RNA extraction buffer (10 mM Tris-HCl, pH 8.0, 1 mM EDTA, 25 mM NaCl) was added. RNA elution was performed rotating overnight at 4°C or on a vortex for 4 h at room temperature. Eluted RNA was separated from the acrylamide gel by centrifugation through a glass fiber filter. The RNA was purified by phenol/chloroform extraction and ethanol precipitation, and the amount of pure RNA was quantified by measuring the absorbance at 260 nm. This RNA pool was used for the first round of selection (= RNA0).

In vitro selection was performed in 100 µl SELEX binding buffer (1x PBS, 1 mM magnesium acetate, pH 7.0) on ice.

First, a counter-selection on Ni^2+^-NTA beads (Promega, MagneHis™ Protein Purification System) was performed in order to remove bead-binding RNAs by equilibration in SELEX binding buffer on ice. Then beads, denatured RNA (500 pmol for the 1^st^ round), and buffer were combined and incubated for 45 min on ice under constant agitation.

A second round of counter-selection was performed in order to remove RNA binding unspecific to proteins. Therefore, His_6_-SNEV (10 pmol protein for the 1^st^ round) was coupled onto the beads in SELEX binding buffer for 15 min on ice. Afterwards, His_6_-SNEV coupled beads were washed with SELEX binding buffer and then the supernatant of the first counter-selection was added and incubated for 45 min on ice as before.

Blom7 wt-His_6_ and Blom7-KH-His_6_ were coupled to the beads (10 pmol protein for the 1^st^ round) and washed several times with SELEX binding buffer. The supernatant of the second counter-selection was added to Blom7 wt or Blom7-KH coupled beads and incubated for 45 min on ice.

Beads of every selection round carrying protein-RNA complexes were washed with SELEX binding buffer, and bound RNA was eluted by addition of nuclease-free water and heating for 1 min to 75°C. The amount of specifically bound RNA was quantified by measuring the absorbance at 260 nm. The eluted RNA was concentrated and finally re-suspended in 10 µl of nuclease-free water.

Afterwards, RNA was reverse-transcribed prior to amplification by M-MLV reverse transcriptase, RNase H Minus, point mutant (Promega). PCR-amplification of the cDNA candidates, and following rounds of selection were performed as before.

Cloning of candidate RNAs

For cloning of candidates the TOPO TA Cloning Kit (Invitrogen) was used. Amplification of the template DNA (2 µl) of selected candidates of the 8^th^ round of SELEX was performed by PCR using AmpliTaq Gold polymerase (Life Technologies). After TOPO-cloning following the manufactures instructions, emerging bacterial colonies were used as templates for a subsequent PCR with primers and polymerase as described above. The PCR products were purified on Sephacryl S-300 (GE Healthcare) spin columns. 200 ng of purified PCR product was used for a subsequent PCR using ABI PRISM BigDye Terminator v1.1 Cycle Sequencing Kit (Life Technologies) and Sanger sequencing. Plasmids of 100 positive clones of each selection were sequenced.

SPR

SPR (surface plasmon resonance) experiments were performed using the BIAcore 3000 (GE Healthcare) system and Sensor Chip CM5 (GE Healthcare). All runs were performed at 23°C.

After equilibration with HEPES buffer (10 mM HEPES, 150 mM NaCl, 50 µM EDTA, 0.005% P20, pH 7.4), the chip surface was activated by applying a mixture of N-ethyl-N'-(3-dimethyl aminopropyl)-carbodiimide hydrochloride (EDC) and N-hydroxysuccinimide (NHS) at a flow rate 5 µl/min with an injected volume of 35 µl.

Proteins were diluted to a concentration of 500 nM in 10 mM sodium acetate, pH 5.0. After immobilization of proteins (flow rate 5 µl/min, injected volume 30 µl), ethanolamine was injected (flow rate 5 µl/min, injected volume 35 µl), the system washed with HEPES buffer, and primed with SELEX binding buffer or HEPES buffer containing 3 mM MgAc. RNAs were denatured, diluted (0.68 – 2 µM) in SELEX binding buffer and injected (flow rate 10 µl/min, injected volume 100 µl). Unspecific binding was considered by subtracting blank runs on a surface not loaded with protein and on a surface loaded with a non RNA-binding control protein. The association and dissociation rates were monitored for 600 s each. Regeneration of the chip surface was performed using 10 mM NaOH.

Immobilization of His6-tagged proteins on CM5 sensor chips was monitored by an increase of response units (RU). We observed that, Blom7α wt showed 5600 RU, streptavidin 3600 RU (as negative control for subtracting unspecific background) and Blom7-KH 3600 RU. As the RU-response is dependent not only on concentration of immobilized agent, but also on the molecular weight, the higher immobilization level of Blom7α was consistent with the higher molecular weight of Blom7α wt (1.5x), compared to Blom7-KH and compared to streptavidin (tetrameric protein, 4x 13 kD).
